# A de novo GABPA Variant in a Patient with Multifocal Cutaneous Vascular Tumors of an Unclassified Entity

**DOI:** 10.64898/2026.09.21.753178

**Published:** 2026-09-25

**Authors:** Annegret Holm, Pascal Brouillard, Nagi Mahammadzade, Mehrnaz Mehrabipour, Anna Shimbulu, Dionyssios Benetatos, Nisha Limaye, Ahmad Alomari, Leonard I. Zon, Whitney Eng, Harry Kozakewich, Friedrich G. Kapp, Miikka Vikkula, John B. Mulliken, Joyce Bischoff

## Abstract

We investigated the genetic basis of a previously unclassified congenital vascular anomaly in a patient with multifocal cutaneous vascular tumors. Through genomic analysis, we identified a novel heterozygous de novo germline variant in GA-binding protein-alpha (GABPA), an ETS family transcription factor. Histopathologic evaluation demonstrated capillary-venous lesions lacking glucose transporter 1 expression, distinguishing this entity from common infantile hemangioma. Notably, the tumor vasculature exhibited a prominent alpha-SMA-positive perivascular cell layer, with nuclear localized GABPA detected in endothelial and perivascular cells. The GABPA c.509T>G (p.L170R) variant is absent from large population databases, including gnomAD, DeCAF, and RGC-MCPS databases, indicating it is extremely rare. The affected leucine residue is highly conserved across species and located within the Pointed (PNT) domain, a critical region mediating GABPA protein-protein interactions. AlphaMissense predicts this substitution to be highly damaging. Structure-based analysis using an AlphaFold-predicted approach, combined with in silico mutagenesis and molecular interaction analyses, revealed that the L170R substitution introduces new electrostatic interactions while disrupting native hydrophobic contacts within the PNT domain. Functional assessment in zebrafish demonstrated that mosaic stromal expression of GABPA-L170R caused abnormal vascular architecture compared with GABPA-wild-type controls, establishing a direct link between this variant and disrupted vascular development. Collectively, the genetic rarity, evolutionary conservation, predicted structural perturbation, and disruptive effects on vascular development establish GABPA-L170R as likely disease-causing variant. These findings define a new molecular mechanism underlying congenital vascular tumor formation and expand the role for ETS-family transcriptional regulation in human vascular anomalies.

## Full Text Availability

The license terms selected by the author(s) for this preprint version do not permit archiving in PMC. The full text is available from the preprint server.

